# Recovering disrupted social capital: insights from Lao DPR rural villagers’ perceptions of local leadership

**DOI:** 10.1186/s12889-016-3858-3

**Published:** 2016-11-25

**Authors:** Jinho Kim, Ji-Hye Kim, Vanphanom Sychareun, Minah Kang

**Affiliations:** 1 Department of Sociology, University of Wisconsin–Madison, 1180 Observatory Drive, Madison, WI 53706 USA; 2 Department of Curriculum and Instruction, University of Wisconsin–Madison, 210 Teacher Education Building 225 North Mills Street, Madison, WI 53706 USA; 3 Faculty of Postgraduate Study, University of Health Sciences, Vientiane, Laos; 4 Department of Public Administration, Ewha Womans University, 11-1 Daehyun-Dong, Seodaemun-Gu, Seoul, 120-750 Korea

**Keywords:** Social capital, Local leadership, Capacity building, Community health, Lao PDR

## Abstract

**Background:**

Social capital is often believed to be one of the key prerequisites for successful implementation of community-based health programs. In less-developed countries, local leaders are positioned as major players in broad community health strategies and interventions, and their capacities and roles are expected to increase in prominence in future community-health-care promotions. In this study, we examined how local leaders’ capacities could be related to social capital in rural villages in Lao PDR, and thus to villagers’ willingness to participate in community-based health efforts.

**Methods:**

We adopted a qualitative approach, conducting semi-structured interviews for both individuals and focus groups. In 2012, 103 people from six villages in the Khoun and Phoukoud districts participated in the interviews. For the individual interviews, we interviewed 22 mothers who had given birth in the past 5 years. For the focus groups, we interviewed 30 women (six groups), 30 men (six groups), and 21 senior villagers (five groups).

**Results:**

First, we noted large variations in the levels of community social capital across villages: four out of six study villages showed a high level of social capital, while two villages suffered greatly from a low level of social capital. In search of the reasons for the disrupted social capital in the latter two villages, interviews revealed that failed leadership, especially in regard to local resource allocations—lack of transparency and corrupt practices—were commonly cited reasons for disrupted social capital. The data also showed that the villagers’ mistrust of these failed local leaders critically reduced their willingness to participate in community-based health efforts, and especially in those that involved resource mobilization and risk-sharing for healthcare. Finally, we found that good communication skills and participatory decision-making styles were attributes that rural villagers in Lao PDR expected of their local leaders.

**Conclusion:**

This study suggests that failed local leadership is detrimental to community participation, resource mobilization, and building communities’ social capital. To achieve intended health care goals through community-based interventions, there is a need to first support local leadership at all levels through capacity-building and improved communication within communities.

## Background

Researchers have documented the potential role of social capital in strengthening the effects of community-level health efforts to improve health outcomes [1, 2]. In this regard, by engaging the whole community in health-promotion efforts, community-based interventions are intended to facilitate collective efficacy within a community, and foster the community’s ownership of and commitment to these health initiatives. Strengthening social capital and community capacity has thus been viewed as one of the key prerequisites for successful implementation of community-based health and development programs [2–4]. While it is critical to gain a better understanding of the determinants of social capital for developing interventions that will boost social capital for purpose of health promotion, very little is yet known about what determines the social capital of a community, and how social capital influences community activities [5].

In particular, the role of local leaders is a potentially profound yet understudied topic that could contribute to our understanding of the relationship between social capital and the success of community health interventions in developing countries. More so than in developed countries, local leaders in less-developed countries are expected to establish bonds and close relationships with members of their communities. This familiarity is often a result of having lived among or near their constituents for an extended period of time [6–9]. In addition, where people are alike in terms of socio-demographic and economic conditions, they are likely to share similar social and cultural identities. Especially in a traditional society, they are influential enough to induce change in the social and cultural behaviors of communities [10]. In this setting, local leaders are in a better position to influence trust, solidarity, and effective community networks among villagers through various types of socio-economic involvements [11].

Despite the clear significance of local leadership, not all local leaders are well-intentioned and capable. They often fail to meet the needs and expectations of their communities, thereby destabilizing trusting relationships with villagers [12, 13]. There is, however, a paucity of research on villagers’ perceptions of such failures in local leadership, and the potential ramifications of these failures for community participation and resource mobilization among villagers.

If community leadership does indeed critically influence community social capital, which is believed to be one of the most important prerequisites for effective community health interventions [1, 14, 15], then merely putting forth larger budgets, advanced technologies, additional training, or technical assistance will not necessarily produce successful outcomes. Efforts to address the problems of leadership failures and weak social capital within a community should precede, or at least parallel, community-based efforts for health care. In the context of less-developed countries with scarce financial and human resources, the influence of local leaders can be harnessed to optimize the use of available resources to improve a community’s social capital.

In this regard, the distinctive roles played by local leaders in rural settings in less-developed countries ought to be better understood, in order to address social and health intervention efforts at the community level. In this study, based on qualitative data obtained through in-depth interviews with local village people in Lao PDR, we first looked into large variations in the level of social capital across rural community settings. We then examined how failed local leadership, as perceived by local villagers, could explain disrupted social capital in certain villages. We further explored how failed local leadership and consequent disruption of social capital could adversely influence community efforts, such as, for example, by negatively affecting villagers’ inclinations to participate in community-based efforts for health care. Lastly, based on interview data, we discussed the desired attributes of good local leadership that are expected by local villagers.

## Methods

This study reanalyzed the data used by Ngan and colleagues [16] and Sychareun and colleagues [17]. The former study focused on the utilization of maternal health care services, and the latter examined constraints experienced by service providers when providing maternal and child health services. The data were collected through semi-structured interviews for individuals and focus groups. To obtain information on health-care-utilization practices, in-depth interviews with women were aimed at understanding the participants’ personal experiences with their local leadership in the context of using health care services, such as whether they received encouragement and access to services for example. Then, focus group interviews were conducted separately for groups of women, men, and the elderly, covering the same range of topics as the individual in-depth interviews, in order to identify the potentially differing perspectives of the different demographic groups.

### Sample and study setting

The data covered six provinces in northern Lao PDR, including four villages (KLN, KLF, KHN, and KHF) in the Khoun district, and two villages (PLN and PHF) in the Phoukoud district. Pseudonyms were used throughout this study. These six villages were selected through theoretical sampling based on ethnic diversity and geographical conditions such as distance to the closest health facility. Hmong is the major ethnicity in KHN, KHF, and PHF, while Lao is the main ethnicity in KLN, KLF, and PLN.

Within the chosen villages, participants were recruited using the convenience sampling method. Interviews took place away from distractions, in private, quiet, and comfortable places, such as a separate room in the village center. Eleven interviewers from a Lao research team at the University of Health Sciences in Lao PDR conducted the individual interviews and focus groups. The semi-structured interview protocol was pre-tested to assess whether it was culturally relevant and acceptable to local participants, that is to say, village community members. The interviewers undertook a 3-day training course prior to data collection in early and mid-January, 2012. Individual interviews took about two and half hours to complete, and focus groups took about 3 hours. As an incentive to participate in the interviews, each interviewee received some financial reward (equivalent to one US dollar) or a small towel.

From February to March 2012, 103 people participated in the interviews. Participants for the individual interviews included 22 mothers who had given birth in the past 5 years, and the mean age of the mothers was 27.9 (*SD* = 6.4), ranging from 21 to 45 years of age. Focus group participants comprised 30 women (in six groups), 30 men (in six groups), and 21 senior villagers (in five groups). The mean age of participants for the focus groups was 39.1 (*SD* = 15.0) (Table 1).

**Table 1:** Study sample of selected villages and respondents

Village^a^	Number of participants (A + B)	Number of individual interviewees (A)	Number of focus groups	Number of focus group participants (B)	Description of Village
KLN	17	5	2^b^	12	Khoun District, Lao-dominated, located close to local health centers
KLF	21	4	3	17	Khoun District, Lao-dominated, located far from local health centers
KHN	17	2	3	15	Khoun District, Hmong-dominated, located close to local health centers
KHF	17	4	3	13	Khoun District, Hmong-dominated, located far from local health centers
PLN	16	4	3	12	Phoukoud District, Lao-dominated, located close to local health centers
PHF	15	3	3	12	Phoukoud District, Hmong-dominated, located far from local health centers
Total	103	22	17	81	

^a^pseudonyms were used

^b^no focus groups for the elderly group were conducted in KLN

### Data analysis

All interviews were conducted using either Lao or Hmong, depending on participants’ language preferences. Audio-recorded responses were transcribed in Lao and Hmong and typed into a computer word-processing program, together with observation notes taken during the interviews. Transcriptions were translated from Lao and Hmong into English, and uploaded into QSR NVivo 10, a data-analysis program for the coding and retrieval of qualitative data. The research team used inductive coding to identify patterns in the data to establish themes [18]. The first round of coding was an open initial coding of the data, with a focus on individual cases of participants. In the second round, the coding was issue-focused [19] and the researchers examined participants’ attitudes toward local leadership and leaders’ roles in community health interventions. Multiple coding processes were carried out based on key phrases, and codes were organized by common categories and sub-categories. While organizing the multiple layers of codes, characteristics of villages and village heads were analyzed and categorized. Regular meetings were held to discuss thematic categories, and to arrive at a list of recurrent themes.

The protocols for individual interviews received institutional review board (IRB) approval from the Ethics Committee for Health Research, at the University of Health Sciences, Lao PDR. The design protocol called for voluntary participation in the interviews, and researchers received participants’ informed consent prior to each interview. Participants were informed of the purpose of the study prior to the interview session, and gave their verbal consent for the interviews to be recorded. They were informed that they could stop the interviews at any time, and were assured of confidentiality.

## Results

### Village-level variations in levels of social capital

First, we present evidence that there are large variations in the level of social capital across rural villages in Lao PDR. To assess the level of social capital present in a village, we follow the structural and cognitive distinction that has gained prominence in the current literature [20]. Structural and cognitive social capital are two mutually reinforcing conceptual dimensions: the structural dimension involves group membership, civic engagement, and social support from the community; the cognitive dimension concerns trust, social harmony, reciprocity, and a sense of belonging [20, 21]. Based on these themes of structural and cognitive social capital, we assess participants’ experiences with, and perceptions of, fellow villagers, to categorize our study villages into two groups: villages with a high level of social capital, and villages with a low level of social capital.

#### Villages with a high level of social capital

Our interview data from the villages of KHF, KLF, KHN, and PLN indicated that the interviewees in these villages conveyed that there was a high level of social capital among the community members. For example, a mother from KHF explained how village members often helped each other:

*In our village, there is a friendship and solidarity among us, such that we can borrow money from each other when we are sick…. Moreover, we provide social and psychological support to each other. When someone is sick, we collect money from neighbors, 20,000 to 30,000 kip each. The neighbors also bring the patients to hospital. (KHF, Hmong, early 20s)*.

The above response shows that the villagers were willing to lend money without any interest, and that they would readily help the sick both financially and psychologically. These mutually beneficial and cooperative behaviors reflect a high level of structural social capital among the villagers.

Similarly, in the other three villages, the villagers would take good care of the sick, willingly help patients’ families by sharing food, cultivating rice fields on behalf of patients’ families, lending money, providing transportation to the hospital, and giving moral support in various forms.

*When there was someone getting sick in a family, the villagers assisted them. For example, assisting in transportation to the hospital. When the sick family did not have enough money, the villagers collected money together. In addition, they also helped with cultivating and harvesting rice. After finishing their own rice field, people assist others to do their rice field, especially the sick families. (KLF, Lao, late 40s)*.

*In the past there was a family that got sick. They vomited and weren’t able to eat, and had diarrhea. People collected money and helped to take them to the district hospital. People in the village did not stop helping them until the sick family got better. (KHN, Hmong, early 30s)*.

*Our people support each other during the cultivation season. We also look after and care about the sick. We make contributions to help them cover medical costs, and visit them after discharging from hospital. (PLN, Lao, late 20s)*.

Not surprisingly, there was evidence that structural and cognitive dimensions of social capital are mutually reinforcing in these four villages: social support among villagers is driven by a sense of trust, reciprocity, and connectedness among villagers, which is in turn strengthened by social support, as noted in the following comment:

*I trust my villagers because they help one another. For example, when I had any troubles, they came to help me all the times, even when I didn’t ask for assistance. (KLF, Lao, early 30s)*.

In general, such a strong sense of connectedness and social support in times of illness and emergencies among community members indicates a high level of social capital, which is essential for successful community-based health efforts.

#### Villages with a low level of social capital

In contrast, our interviews indicate that, among the six villages, villagers from the two villages of KLN and PHF were experiencing symptoms of a low level of social capital. In those villages, interviewees reported that they were not willing to engage in civic activities such as communal works and village meetings. For example, a village participant from KLN said that people in her village possessed no solidarity and did not want to engage in any civic activities that required community participation.

*People in our village do not have solidarity. For example, they do not share labor and they do not attend village meetings. (KLN, Lao, early 30s)*.

Given that the agricultural environment of rural Lao PDR requires a lot of collective labor and shared use of public facilities and resources [22], such poor civic engagement may reduce agricultural productivity, and offer more cause for concern about means to implement viable community health programs. Similarly, a participant from PHF responded to the question on whether she could be expected to get help from her village members as follows:

*No, if there is any problem, they will not help, and if we would like to borrow money, they will not help either. Someone might have money but they will not give you any, even as a loan. When we don’t have rice, they will help only a little…. (PHF, Hmong, early 30s)*.

In addition to low levels of structural social capital, the data suggest that the two villages, KLN and PHF, suffer from disrupted cognitive social capital, namely, lack of trust, solidarity, and a sense of belonging among villagers. The following interview response showed that a lack of solidarity existed among villagers:

*During the flooding in 2011, there was nobody to help me. I cried as my house, rice, and other things were flooded. I called for help, but nobody came to help me…the villagers did not have any friendship or solidarity among themselves; the villagers hated each other. (KLN, Lao, mid 20s)*.

According to this participant, distrust and animosity were prevalent among the villagers. In addition, villagers described their neighbors as “insincere” and “without solidarity.” Furthermore, a woman from KLN said that due to the lack of community solidarity in her village, villagers she knew would not be willing to participate in the much-needed mother-and-child health programs:

*I think it is difficult to motivate them to actively participate in development programs, including mother and child health programs, since they have no spirit of solidarity. (KLN, Lao, early 30s)*.

The above responses illustrate that these villagers harbored high levels of mistrust, and maintained attitudes of watching out only for individuals’ self-interest. This situation would only lead to low demand for community-based health programs, especially in scenarios when resource mobilization and risk-sharing are deemed necessary. Villagers would not benefit from utilizing social networks within their community in times of need. They would be unwilling to help each other and share labor or resources, both of which are common practices for the sustainable existence of most rural agricultural communities in Lao PDR [22].

### Failed leadership and disrupted social capital

We now turn to an examination of the potential factors that might lead to different levels of social capital across villages. In particular, the study focuses on the roles of local leadership. Interview data revealed that participants from the villages of KLN and PHF (characterized by disrupted social capital) perceived failed village leadership, and described how these leaders affected social capital in their villages, which in turn precipitated their unwillingness to participate in community-based health efforts such as a village fund. Local leaders’ failures in the two villages of KLN and PHF took various forms, but embezzlement and favoritism were the most commonly mentioned causes.

#### Embezzlement

Notably, corruption scandals involving village leaders are probably the most serious barrier to creating social cohesion and trust within communities. In particular, interview participants in KLN and PHF reported many forms of corruption, which included fraud, abuse, or mismanagement of public resources. A participant from KLN village explained in detail how several instances of corruption led her to mistrust her village head. For example, she described the village head’s embezzlement of government aid and company compensation funds intended for her family:

*I do not like the village head. For example, hydropower equipment was to be constructed in my paddy field. The electric company said to pay for compensation of nineteen million kips…after arriving home and checking the money that the village head delivered, we found out that we got only nine million kips….The village head told us that he would ask about it and get the remaining money for us back, but we have not received anything yet. (KLN, Lao, early 30s)*.

In addition to embezzling external aid and compensation funds, the same village head was also thought to have mismanaged community budgets:

*When there was flooding in our village in 2011, the province provided money to the village. The village head went to get money at the provincial office, but he did not give that money to people. And, when assistance with relief supplies came from the province, the village head gave them only to his relatives. (KLN, Lao, early 30s)*.

A participant from PHF reported a series of corrupt practices by a local leader, which explained why the village head lost the trust of villagers in that community:

*One case concerning the rice bank: people kept rice in the village’s rice bank, but when they needed rice back, they could not get it and rice disappeared. Another story: when officials from the province visited our village, the village head asked each household for 50,000 kips to buy goats for preparing a welcome party for those guests, but villagers never saw any goats during the party. Most of the villagers did not trust the village head. (PHF, Hmong, early 30s)*.

#### Nepotism and favoritism

Villagers’ perceptions of local leaders’ practices that leaned towards nepotism or favoritism and other biased acts were prevalent across the villages of KLN and PHF. The data revealed that the nepotism of village heads manifests itself in two forms: granting decision-making power to selected groups, and unfair distribution of limited resources. First, the village head of KLN granted family members and close relatives political power to make important decisions on behalf of the community. One community member of KLN explained what she witnessed:

*Mostly, the village head tries to involve people that he knows and he is familiar with. For example, the head of the Lao Women’s Union (LWU) is a relative of the village head. (KLN, Lao, mid 20s)*.

In some villages, village heads’ relatives were selected as the main beneficiaries of financial and medical aid, despite their lack of eligibility for these benefits. At the time of the interviews, five out of six study villages managed health equity funds, and three villages had village funds. Some communities ran their own projects, such as a rice bank and animal funds, supported by external development agencies. Our interviews revealed that many villagers from KLN and PHF witnessed significant preferentialism toward the relatives of village heads during beneficiary selection processes. For example,

*The village head selected his relatives and people who work with him…the village head made decisions based on his own interests and for his closest relations without any consideration for the poorest families…I know there were seven poor families in the village. (KLN, Lao, early 30s)*.

In the village of PHF, there were similar claims that the village head’s family members received the most benefits from health care programs, while more needy candidates were excluded. Besides this preferential beneficiary selection, several participants reported that their deputy village head claimed the most arable banana field, and then distributed less arable areas to other villagers. Similarly, it was reported that in serving his family’s interests, the village head unfairly acquired the rice field closest to the village, while others were allocated rice fields far from their homes.

#### Failed leadership and community health promotion efforts

As shown above, in KLN and PHF, many villagers expressed anger and disappointment with what they perceived to be corrupt and unfair decision-making by their village leaders. More notably, where there is suspicion and hostility toward local leaders, there is also a tendency toward discontent among village members, and distrust toward new practices proposed by their local leaders. In these contexts, the fair use of funds and transparent beneficiary selection processes were seen as being significant, not only because money or in-kind transfers were involved, but also because villagers’ negative experiences were likely to adversely influence their willingness to participate in future community activities.

The data show that in the KLN and PHF villages, villagers seemed to be notably pessimistic about community-based funds for health interventions. The village heads in these two villages seemed to have lost the villagers’ confidence that they could manage community resources and treat all villagers fairly, so the villagers were not convinced they should be involved in such community-based efforts.

*I think it is possible to have a village fund in our village to help people who are sick, only if we get a new village head whom we can trust and who is sincere. The villagers don’t dare to give more money because they don’t trust the village head anymore since we don’t know what the village head will spend money on. (KLN, Lao, mid 20s)*.

Such corrupt practices by village heads posed the biggest obstacle to mobilizing community members and optimizing the use of collective resources. A respondent from PHF village reported that their village leaders did not properly manage a rice bank program, which they believed reduced trust toward village authorities:

*A village revolving fund would be good because people could borrow money to spend on health care or buying cattle. However, we are scared of anything that looks like the revolving rice fund that we once had. The village leader who was responsible for the revolving rice fund didn’t record accurately about who had borrowed rice….After that, none of the villagers trusted him any more. (PHF, elderly focus groups)*.

Though a majority of villagers agreed on the need for a new village fund to help the poor and the sick, their previous negative and disappointing experiences with how their village authorities managed the rice fund made them wary, and reluctant to participate in new community-based funds.

### Expected attributes for successful local leadership

While the two villages of KLN and PHF were reported to have suffered the most from the failures in the leadership of their village heads, the other four villages maintained relatively stable and trusting relationships with local leaders. Local leaders in these villages can be characterized as having the following attributes.

#### Capability of effective communication

Local leaders are expected to be effective communication channels through which external information enters and spreads throughout a community, so that villagers may benefit from access to this information. When a health staff member from the province or district approaches a village, they first provide information to the local leaders (i.e., a village head) and this leader is expected to disseminate the information to the village. To introduce a new community project, local leaders are expected to hold information sessions to both disseminate information and receive feedback. At community meetings such as a town hall meeting for example, details of a new project are communicated to members; at the same time, members’ voiced concerns are heard, and suggestions are made. Some village leaders were perceived as capable of delivering on their roles as effective information sources or channels:

*He is good at sharing information…he immediately informs people of whatever he receives, for instance, information about immunizations. (KHF, Lao, early 20s)*.

*We have a good village head because he always announces when we have a meeting. For example, when health staff visits our village, he encourages us to bring our children to the village hall. (PLN, Lao, late 20s)*.

In contrast, villagers from the two villages with failed local leadership pointed out that the low level of participation in civic activities and health campaigns may also come from poor communication. An interview response from KLN indicated that the village head was not an effective communicator:

*I never participated in village activities because I did not know or hear, and was not aware of the village activities. When I asked other people about the plan for village activities, they didn’t know either. Nobody told us about them. (KLN, Lao, mid 20s)*.

This respondent complained that she never participated in community activities because neither her neighbors nor the village head had ever notified her that these meetings would be taking place. In this regard, the village leader failed to have the attributes of successful local leadership in terms of communication.

#### Participatory decision-making

Hierarchical relationships between local leadership and village members can create tension and conflict when there are competing interests such as the selection of recipients for financial or food aid. In these instances, villagers are understandably interested in knowing how local authorities and leadership committees make these important decisions.

Village participants from KHF perceived that they had a good relationship with their village head, and seemed to be satisfied with the way their leaders made decisions on behalf of the villagers. They reported that they were fully informed of the criteria for beneficiary selection, and believed that the village funds that had been allocated had helped needy villagers in their community:

*We are happy with the village head’s government. When we had conflict and quarrels, he was able to negotiate: for example, recently when we weren’t able to finish harvesting on time, he consulted with committee members to find the way to solve this problem…he requested the youth in the village to help finish harvesting. (KHF, Hmong, early 20s)*.

*For the health equity fund, all villagers join to select families who have no property such as paddy fields and cattle. (KHF, Lao, early 20s)*.

Villagers from KLF also reported that their village head was eager to encourage village members to participate in making a decision about village affairs.

*When a water pipe in the village was stuck, he called all villagers to repair it together. In case of having a project coming, he also called all villagers to come for a meeting, informed them about the project, and asked them whether they agree to have it. (KLF, Lao, early 20s)*.

*In our village, we now have a village fund. Before introducing the project, to promote community participation, the village leader gathered the villagers together to inform about the project and asked their opinions about the project. (KLF, Lao, late 20s)*.

In contrast, in the two villages with failed local leadership, villagers were excluded from important decision-making processes or were not informed properly of decisions that concern them, so they doubted the sincerity and leadership of their village heads:

*The village head and his committee decided who they would give money as loans, and then informed us later who they gave money to. I want the village head to discuss with the villagers before decisions like this are made. (KLN, Lao, late 30s)*.

*I would like to know everything and would like to have people join decision-making process in all its aspects. (PHF, Hmong, early 30s)*.

In their mistrust of the selection process for beneficiaries of the village funds, study participants in KLN and PHF questioned their leaders’ intentions, wanted to know whether the process was rational and fair, and also showed a keen interest in participating in this decision-making process.

## Discussion

With the goal of better understanding how social capital can be improved and transformed into community capacity, our study focused on the role of the local leadership in rural community settings in Lao PDR. In many less-developed, local leaders such as village heads, elders, and traditional authorities in Lao PDR are often key decision-makers for community development and health efforts [8, 9, 12, 23]. Especially village heads—who are elected every 3 years by receiving the highest number of votes among five candidates who were screened by district Party officials and the Governor’s office—can be influential enough to induce changes in the social and cultural behaviors of communities [10, 24]. Village heads are responsible for various administrative duties that include but are not limited to “solving conflicts, keeping village records, facilitating local development efforts, and more recently collecting taxes” [24]. Based on qualitative interviews with villagers, we were able to identify areas and ways in which local leaders were perceived to have failed to fulfill their responsibilities. We also explored how such failures affected community social capital in terms of villagers’ acceptance of, and willingness to participate in, community-based health programs.

Our findings can be summarized and discussed as follows: First, from our interview data, we noted significant variations in the levels of social capital across villages. Interviewees from two villages commonly and persistently conveyed the lack of social capital, whereas in the remaining four villages we observed evidence of not only a strong sense of social connectedness, but also social support among villagers. In the villages with a high level of social capital, villagers were very willing to help each other by providing both psychological and physical support for the poor and the sick. We also found that these villages benefit from high levels of cognitive social capital among villagers, such as trust, reciprocity, and a sense of connectedness [25]. In contrast, in the villages with a low level of social capital, we observed villagers’ unwillingness to help each other in times of need, which in turn could lead to psychological distress and increased risk of poor physical health among villagers [26–29]. Pregnant women and children are probably those most vulnerable to this lack of solidarity and reciprocity among villagers, because their health and wellbeing are known to benefit from the village communities’ social support [30].

Secondly, interviews revealed that failed leadership was a commonly cited reason for disrupted social capital. While most villages in Lao PDR have Conflict Resolution Committees (*khai khia*) to resolve civil disputes among villagers (e.g., land disputes, marital disputes, and petty crimes), there are no formalized systems to address village heads’ poor administration and/or bad leadership [24]. Frequently, failed local leadership due to a lack of transparency, as well as corrupt practices in local resource allocations were identified in this study. Many interview participants from the villages with failed leadership vividly reported specific corruption practices in which their village heads are allegedly involved. Moreover, village heads’ unfair practices were prevalent in the villages during the process of selecting beneficiaries of community-based programs that involved financial and medical benefits for the sick. This finding is also consistent with prior literature that underscores the significance of local leaders as a critical component of community capacity [31, 32] and sources of social capital [33]. Our results also support the view that local leaders are in a position to directly and indirectly influence trust, solidarity, and effective community networks among villagers through their involvement in the social and political life of their community [11].

Of greater importance, we observed evidence of poor mobilization for potential community-based health interventions as a result of failure in local leadership in the villages with a low level of social capital. The data showed that when villagers perceived their leaders as being corrupt, their mistrust reduced their willingness to participate in the programs involving resource mobilization and risk-sharing, such as village revolving funds and community-based health insurance. This result is consistent with previous studies that found community-level social capital (e.g., strong trust-based relationships among their community members) is a crucial determinant of the success of community-based health insurance programs [1, 14, 15]. However, our findings extend prior research by demonstrating that local leadership failure, as perceived by villagers, impedes uptake and effective implementation of community-based health interventions.

Finally, our interviews revealed Lao PDR villagers’ expectations that their leaders should have good communication skills and participatory decision-making styles. Our findings lead us to understand that in capacity building, an important skill or capability of local leaders is not only to communicate openly and frequently, but also to invite community members to voice and share their opinions. Despite the potential for, and prominence of, this role, local leaders do not always meet their communities’ expectations. Local leaders who adopt authoritarian and patriarchal approaches are viewed as barriers to improved community health: the “dominance of one leader” can lead to manipulation of the distribution of resources and benefits within communities [23].

### Limitations

Although this study provided valuable insights into the role local leadership plays in community participation and resource mobilization for community-level health efforts and building community social capital, some limitations should be noted. First, the voluntary nature of the interviews may exacerbate possible issues related to self-selection into the study population, which could bias the results of the study. The extent to which the findings can be generalized will be limited by the fact that the sample population was not randomly selected, or nationally representative. Similarly, since a majority of the participants were women with children and elderly men and women, readers should keep this sample restriction in mind when interpreting the results. Second, despite the study’s decent sample size, all representative opinions or themes may not have emerged from the data since sampling did not continue until informational redundancy or saturation was achieved [34, 35]. Third, this study focuses on villagers’ perceptions of local leadership and how these perceptions affect their willingness to join community-based health efforts; it does not take into account the views of the local leadership, though we interviewed them. While it is possible that the local leadership would have different views on their leadership experiences and capabilities, we relied on villagers’ responses because we viewed the villagers’ *perceptions*, rather than local leadership’s *actual* performance, as the determinant of acceptance of, and willingness to participate in, community-based health programs.

## Conclusions

The findings of this study could be policy-relevant. While community-based health interventions should include measures to encourage the community’s active participation, this study suggests that these interventions must be accompanied by efforts to address governance concerns regarding expectations for good local leadership. Failures of local leadership are detrimental to the successful implementation of community-based health efforts, especially when those leaders are entrusted with mobilizing, pooling, allocating, managing, and supervising health-care resources [13, 36–38].

In order to achieve intended health-care goals through well-received community-based interventions, there is a need, therefore, to support local leadership through capacity building, by establishing mutually-trusting relationships and enhanced communication within communities. One fundamental step might be to promote the sound and transparent management of village affairs, by having policy makers involve the entire community in decision-making processes, and especially in the allocation and management of pooled or public financial resources. In doing so, the roles of less-trusted local leaders would be limited, while whole-community participation can be enhanced.

To conclude, this study provides evidence that failed or weak local leaders discourage community mobilization, which is an essential ingredient of successful implementation of community-based health efforts [20, 21]. In this regard, this study serves as an important reminder to policy makers, that successful implementation of community health intervention programs depends on the capacity of local leadership as well as villagers. If the prerequisite social capital at the community level cannot be activated, new community health programs aimed at promoting community participation and mobilizing local resources are not likely to be effective.

## Abbreviations

Lao PDR: Lao People’s Democratic Republic
SD: Standard deviation
